# A rare anastomosis between the common hepatic artery and the superior mesenteric artery: a case report

**DOI:** 10.1007/s00276-017-1859-2

**Published:** 2017-04-21

**Authors:** Łukasz Olewnik, Grzegorz Wysiadecki, Michał Polguj, Mirosław Topol

**Affiliations:** 1 Department of Normal and Clinical Anatomy, Interfaculty Chair of Anatomy and Histology, Medical University of Lodz, ul. Narutowicza 60, 90-136 Lodz, Poland; 2 Department of Angiology, Medical University of Lodz, Lodz, Poland

**Keywords:** Anastomosis, Common hepatic artery, Superior mesenteric artery

## Abstract

For decades, anastomoses between unpaired branches of the abdominal aorta have attracted the attention of anatomists, surgeons and radiologists, due to their significance in many clinical procedures. This report presents a rare anastomosis between the common hepatic artery and the superior mesenteric artery, which gave off three branches to the jejunum. The diameter of the anastomosis measured at the point of its branching off the common hepatic artery and at the level of union with the superior mesenteric artery was 4.46 and 4.19 mm, respectively. Moreover, the anastomosis gave off the branch to the head of the pancreas. Both embryological background and potential clinical implications of this variation are discussed. Knowledge of these vascular connections may be important for diagnostic and surgical procedures.

## Introduction

For decades, anastomoses between unpaired branches of the abdominal aorta have attracted the attention of anatomists, surgeons and radiologists, due to their prominent significance in many clinical procedures such as surgery of aneurysms or radiological transarterial chemoembolization procedures for tumors [1–4]. The common hepatic artery (CHA) is a short blood vessel that supplies the liver, the pylorus, the pancreas and the duodenum [5]. It arises from the coeliac trunk and divides into the gastroduodenal artery (GDA) and the proper hepatic artery (PHA) [1, 6]. The superior mesenteric artery (SMA) arises from the anterior surface of the abdominal aorta, 1–2 cm lower than the celiac trunk, and gives off five sets of branches: the inferior pancreaticoduodenal artery, the intestinal arteries, the ileocolic artery, the right colic artery and the middle colic artery [5]. The SMA supplies the lower part of the duodenum, the jejunum, the ileum, the cecum, the appendix, the ascending colon and two third of the transverse colon, as well as the pancreas [5].

Several anatomic variations of the celiac trunk, superior mesenteric artery, the right and left hepatic arteries, and the accessory hepatic artery have been described [2, 7–15]. However, few reports exist concerning the variants of the common hepatic artery [16]. This case report describes a rare anastomosis between the common hepatic artery and the superior mesenteric artery. It highlights the importance of knowledge of the arterial supply in the abdominal cavity.

## Case report

**Fig. 1:**
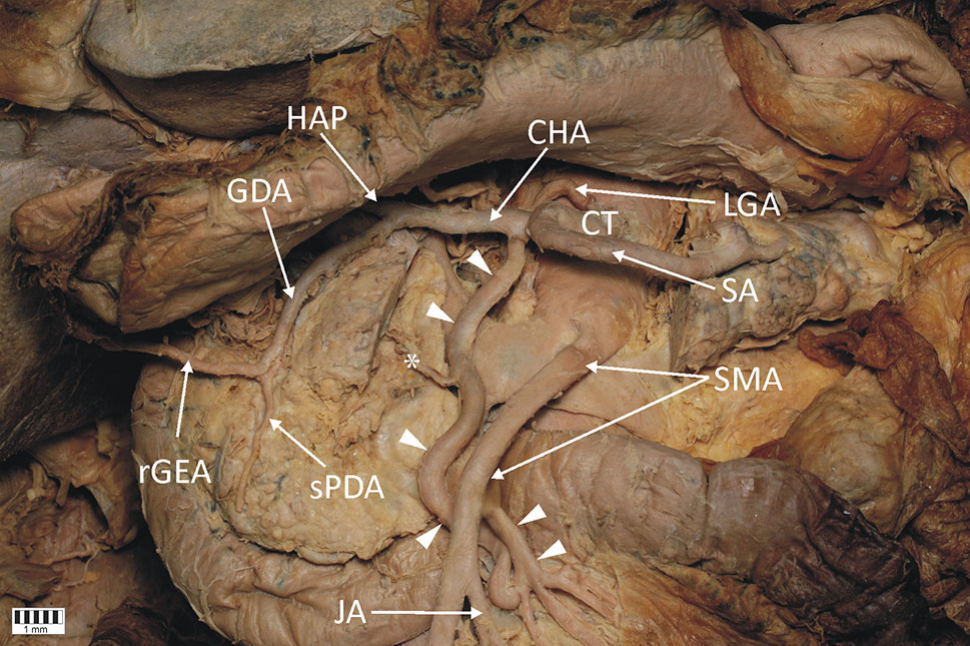
Anastomosis between the common hepatic artery and the superior mesenteric artery (*white arrowheads* present the anastomosis). *L* liver, *S* stomach, *P* pancreas, *CT* coeliac trunk, *SMA* superior mesenteric artery, *LGA* left gastric artery, *CHA* common hepatic artery, *SA* splenic artery, *HAP* hepatic proper artery, *GDA* gastroduodenal artery, *rGEA* right gastroomental artery, *sPDA* superior pancreaticoduodenal artery, *JA* jejunal artery, *bJA* branches to the jejunum. *Asterisk* branch to the head of the pancreas

**Fig. 2:**
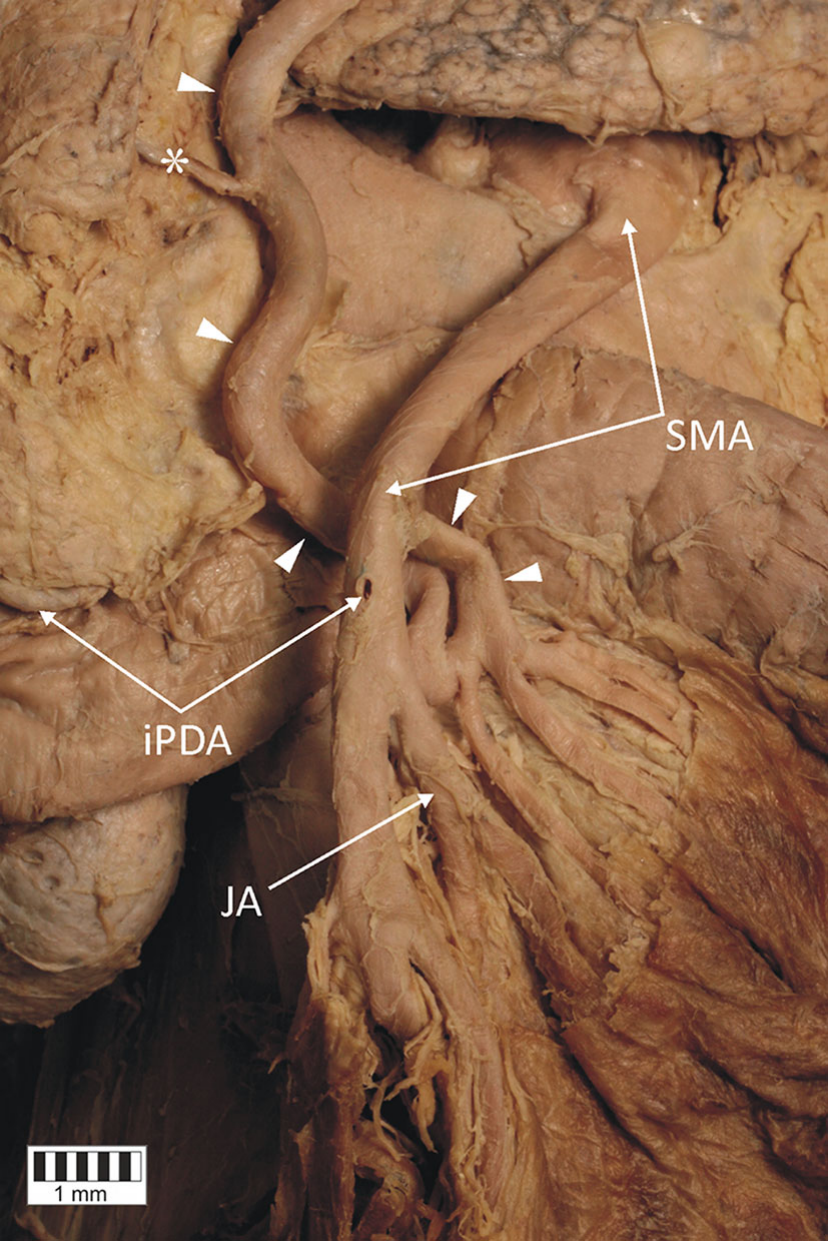
Anastomosis. View of the branches for the jejunum and the connection with the superior mesenteric artery. *White arrowheads* present the anastomosis between the common hepatic artery and the superior mesenteric artery. *Asterisk* branch for the head of the pancreas. *P* pancreas, *SMA* superior mesenteric artery, *iPDA* inferior pancreaticoduodenal artery, *JA* jejunal artery, *bJA* branches for the jejunum

**Fig. 3:**
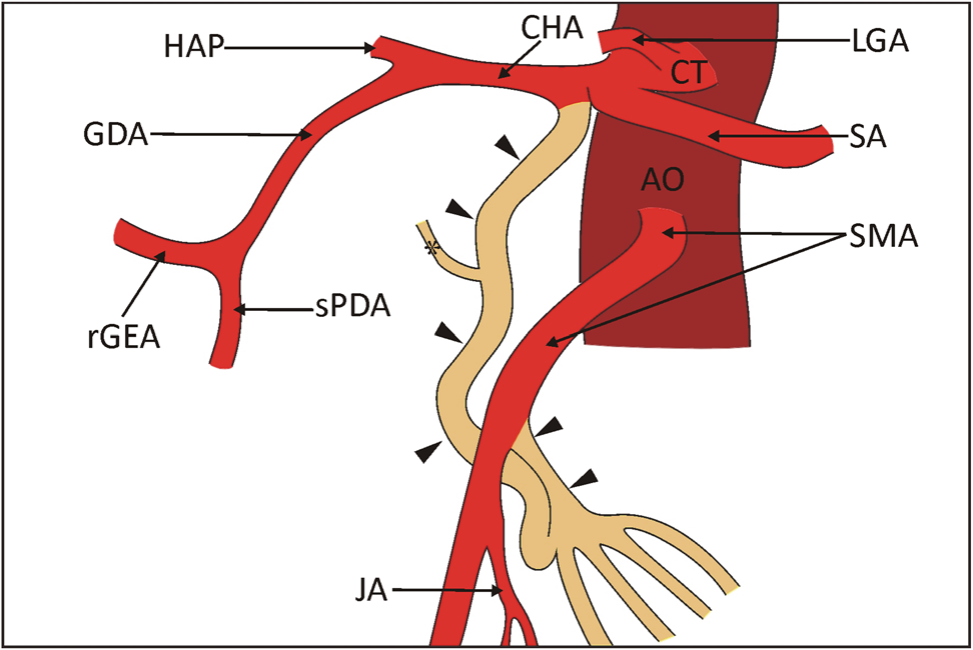
Schematic drawing of the structures of the anastomosis between the superior mesenteric artery and the common hepatic artery. *Black arrowheads* indicate the anastomosis. *CT* coeliac trunk, *LGA* left gastric artery, *CHA* common hepatic artery, *SA* splenic artery, *HAP* hepatic proper artery, *GDA* gastroduodenal artery, *rGEA* right gastroomental artery, *sPDA* superior gastroduodenal artery, *AO* abdominal aorta, *SMA* superior mesenteric artery, *JA* jejunal artery. *Asterisk* branch to the head of the pancreas

The cadaver of a 58-year-old woman was subjected to routine anatomical dissection for research and teaching purposes at the Department of Normal and Clinical Anatomy of the Medical University of Lodz. The dissection was performed in the abdominal cavity. After a careful resection of the interrupting tissues, a rare anastomosis between the common hepatic artery and the superior mesenteric artery was observed (Figs. 1, 2, 3).

The measurements of the arteries were taken from digital photographic documentation processed through MultiScanBase 18.03 software (Computer Scanning System II, Warsaw, Poland). The value and precision of this method have been confirmed in a previous study [10]. A detailed analysis found that the anastomosing vessel followed a spiral route. A loop connection with the superior mesenteric artery was observed in the distal part of this artery, and the branches of the jejunum (diameters: 3.11/2.25/1.81 mm) rooted from the convex part of this connection (Fig. 2). The main arterial trunk of the jejunum branched off below the anastomosis site with a diameter of 3.36 mm (Fig. 2). Furthermore, it was observed that the branch ran from the said artery towards the head of the pancreas (Figs. 1, 2). The main trunk of the anastomosis was 4.46 mm in diameter at the common hepatic artery and 4.19 mm at the superior mesenteric artery. The external diameter of the cross point of the common hepatic artery and the superior mesenteric artery was 4.55 mm.

## Discussion

Since the coeliac vascularization is complex, its anatomical variations should be known by hepatic surgeon during transplant procedure. There are several classifications regarding the origin and topography of abdominal arteries [7, 13, 17–21]. The most common are various types of the celiac trunk branches [6–9, 14, 17, 22], atypical liver vascularization [3, 17, 20] and differences in the territory of the mesenteric arteries [8, 14, 17, 23]. The typical ‘natural’ anastomosis between the superior pancreaticoduodenal artery and the inferior pancreaticoduodenal artery forms an arterial communication named the anterior and posterior pancreaticoduodenal arcade [15, 22, 24].

The variants of the celiac trunk and the superior mesenteric artery are thought to have an embryological basis. Developmentally, the superior mesenteric artery is considered to be a part of the celiac complex, and therefore, variants of the SMA are connected to a large extent on the CT [25]. Tandler [25] suggests that a ‘longitudinal anastomosis’ unites the roots of the ventral segmental arteries. Several anatomical variants of the unpaired arteries of the abdominal aorta develop, depending on the extent of resorption or retention of different parts of the longitudinal anastomosis and ventral segmental roots. Accordingly, the 10th primitive root of the ventral segmental artery becomes the left gastric artery; the 11th becomes the splenic artery; the 12th becomes the common hepatic artery; the 13th becomes the superior mesenteric artery, and the 19th primitive root becomes the inferior mesenteric artery with separate origins from the abdominal aorta [9, 18, 25]. While the celiac trunk is formed by the fusion of first three roots and becomes separated from the fourth root, the superior mesenteric artery develops from the fourth root, which later migrates caudally with the ventral migration of the gut [13]. It is likely that incomplete fusion or malfusion of these arteries during the developmental stage may be responsible for the anastomosis observed in this study.

Lipschutz [21] first classified the celiac trunk in 1917, describing its four variants. In 1928, Adachi [17] also proposed a detailed classification of this region, defining the left gastric artery, splenic artery, common hepatic artery and superior mesenteric artery as the principal branches in this region. He also noted the existence of accessory hepatic arteries, classifying them into six types with 28 forms. Song et al. [26] classified 13 types of the celiac trunk in their studies. Some variants of the coeliac trunk were also described as case reports. Hirai et al. [8] described a case where two trunks replaced the celiac trunk: the hepato-spleno-mesenteric trunk and the gastro-phrenic trunk. Yan et al. [14] reported the celiac and superior mesenteric arteries arising with a common short trunk from the abdominal aorta. Iacob et al. [9] described an absence of the celiac trunk, with the left gastric and the common hepatic arteries originating directly from the anterior wall of the abdominal aorta. Çiçekcibaşi et al. [7] described a rare variation of the celiac trunk which gave rise to six arteries: the left gastric, common hepatic, splenic, left gastroepiploic, and the right and left inferior phrenic arteries.

Tandler [25] and Buhler [27] as first observed and described anastomosis between the celiac axis and the superior mesenteric artery. Buhler was a first, who described the retropancreatic anastomosis between the celiac trunk and middle colon artery [27]. Connection between the branches of the CT and the SMA include the pancreaticoduodenal arcades described by do Rio-Branco on the right side [28] and the Buhler arcade on the left side [27]. This “normal” SMA morphology may be present in as many as 68% of cases [29].

Despite having a normal or variable origin, the common hepatic artery may follow an unusual course. Yan et al. [14] found the artery to have a 13% probability of originating from the superior mesenteric artery. Okada et al. [30] observed the possibility of the common hepatic artery originating from the left gastric artery. Wang et al. [16] described the common hepatic artery originating from the celiac trunk, crossing the portal vein and positioning itself at the back of this structure. From the clinical point of view it is important to establish whether the numerous variants of the vascularization of the abdomen described above play a role in the formation of collateral circulation.

Negovanovic [31] describes the presence of an anastomosis between the common hepatic artery and the superior mesenteric artery in an adult female cadaver. This fusion directly connected the CHA and the SMA [31]. However, our case is quite different, insofar that the ‘connection trunk’ gave one branch to the pancreas and three branches to the jejunum. According to Rosenblum et al. [32], the sources of collateral circulation between the mesenteric and non-mesenteric arteries are numerous and clinically important. Such collateral of the circulatory system can be seen between the celiac trunk and the superior mesenteric artery, and between the superior mesenteric artery and inferior mesenteric artery [32]. The most common potential collateral flow paths between the CT and the SMA comprise the gastroduodenal artery and pancreaticoduodenal arteries [32]. An anastomosis known as the arc of Barkow may be present between the epiploic arteries of the splenic artery and the superior mesenteric artery [33–35]. A rare connection is the arc of Buhler [34, 36]. The presence of the arc of Buhler is on the level of 3.3% [34, 36, 37].

The connection described in this case is clinically important because three jejunal arteries arise from its distal part. Between these three arteries and the first jejunal artery arise several coexisting connections from the superior mesenteric artery. These connections may prevent the occurrence of mesenteric acute ischemia; in case of SMA occlusion. The arterial supply of the jejunum will be provided by this anastomosis, irrespective of the degree of SMA occlusion. As acute mesenteric ischemia accounts for 60–80% of all cases of mesenteric ischemia, and has a mortality rate between 59 and 93% [38–40], knowledge of such anatomical variation is important.

## Conclusion

In conclusion, although the anastomosis between the common hepatic artery and the superior mesenteric artery presented in the case is very rare, it might be a highly significant factor in the arterial supply to this region. Preoperative knowledge of such rare anatomic variants is essential in planning some surgical procedures and liver transplantation.
